# 2-Arachidonoylglycerol Reduces the Production of Interferon-Gamma in T Lymphocytes from Patients with Systemic Lupus Erythematosus

**DOI:** 10.3390/biomedicines10071675

**Published:** 2022-07-12

**Authors:** Luca Navarini, Marta Vomero, Stefano Di Donato, Damiano Currado, Onorina Berardicurti, Annalisa Marino, Pietro Bearzi, Alice Biaggi, Matteo Ferrito, Piero Ruscitti, Marina Fava, Alessandro Leuti, Paola Cipriani, Mauro Maccarrone, Roberto Giacomelli

**Affiliations:** 1 Rheumatology, Immunology, and Clinical Medicine Research Unit, Department of Medicine, Campus Bio-Medico University, 00128 Rome, Italy; m.vomero@unicampus.it (M.V.); s.didonato@unicampus.it (S.D.D.); d.currado@unicampus.it (D.C.); berardicurtio@libero.it (O.B.); a.marino@unicampus.it (A.M.); pietro.bearzi@unicampus.it (P.B.); a.biaggi@unicampus.it (A.B.); matteo.ferrito@gmail.com (M.F.); r.giacomelli@policlinicocampus.it (R.G.); 2 Immunorheumatology Unit, Fondazione Policlinico Universitario Campus Bio-Medico, 00128 Roma, Italy; 3 Department of Clinical Sciences and Community Health, Division of Clinical Rheumatology, ASST Istituto Gaetano Pini–CTO, University of Milan, 20122 Milan, Italy; 4 Department of Biotechnological and Applied Clinical Sciences, Rheumatology Unit, Università Degli Studi Dell’Aquila, 67100 L’Aquila, Italy; piero.ruscitti@univaq.it (P.R.); paola.cipriani@univaq.it (P.C.); 5 European Center for Brain Research (CERC)/Santa Lucia Foundation IRCCS, 00143 Rome, Italy; m.fava@unicampus.it (M.F.); a.leuti@unicampus.it (A.L.); m.maccarrone@univaq.it (M.M.); 6 Department of Medicine, Campus Bio-Medico University of Rome, 00128 Rome, Italy; 7 Department of Biotechnological and Applied Clinical Sciences, University of L’Aquila, 67100 L’Aquila, Italy

**Keywords:** systemic lupus erythematosus, endocannabinoids, 2-arachidonoylglycerol, T cell, inflammation, interferon-ɣ

## Abstract

*Background*: the endocannabinoid 2-arachidonoylglycerol (2-AG) plays a pivotal role in immune cells regulation. The plasma levels of 2-AG are increased in patients with systemic lupus erythematosus (SLE) and correlate with disease activity. Moreover, in plasmacytoid dendritic cells from SLE patients, 2-AG is able to control the production of type 1 interferon (IFN) through CB_2_ activation. The aim of this study was to evaluate the potential role of 2-AG on T lymphocytes from SLE patients. *Methods*: peripheral blood mononuclear cells (PBMCs) from SLE participants and age- and sex-matched healthy donors (HD) were isolated by Ficoll–Hypaque density-gradient centrifugation. The PBMCs were treated with increasing concentrations of 2-AG, and AM251 and AM630 were used to antagonize CB_1_ and CB_2,_ respectively. Flow cytometry was used to assess the expression of CD3, CD4, CD8, CD25, IFN-ɣ, IL-4, and IL-17A. *Results*: 2-AG (1 μM) decreased IFN-ɣ expression (*p* = 0.0005) in the Th1 lymphocytes of SLE patients. 2-AG did not modulate the cytokine expression of any other T lymphocyte population from either SLE or HD. Treatment with both 2-AG and AM630 increased the IFN-ɣ expression in Th1 lymphocytes of SLE patients (*p* = 0.03). *Discussion*: 2-AG is able to modulate type 2 IFN production from CD4+ T lymphocytes from SLE patients through CB_2_ activation.

## 1. Introduction

Systemic lupus erythematosus (SLE) is a chronic autoimmune disease potentially involving multiple organs [1]. SLE treatment is still a challenge for clinicians, and patient-tailored interventions to manage disease activity and slowing damage accrual are not completely established [2,3,4,5]. Despite the advances in understanding the role of genetic polymorphisms, alterations in the innate and adaptive immunity, together with environmental factors, may also contribute to disease initiation and progression. Yet, this complex interplay has not been completely understood [6].

In this arena, growing evidence has shown that alterations in lipid mediators’ balance and metabolism may play a pivotal role in the pathogenesis of SLE [7]. Higher blood levels of low-density lipoproteins (LDL) and high-density lipoproteins (HDL) are common in SLE patients when compared to the population at large and, along with chronic inflammation, disease activity, and sedentary behavior, represent a causative factor of accelerated atherosclerosis [8,9,10,11,12]. Furthermore, lipids play several important roles during immune response in SLE, switching or alternatively turning off inflammation, and modulating macrophages’ activation, leading to pro-inflammatory cytokines production [7,12,13].

Furthermore, T and B cell membranes of SLE patients are richer in cholesterol and glycosphingolipids, leading to alterations in lipid rafts and dysregulation regarding cell signaling, thus influencing the normal regulatory T (Treg) lymphocytes/T helper 17 (Th17) lymphocytes ratio [14,15,16,17,18,19,20].

Endocannabinoids (eCBs) seem to play an important role in this lipidomic dysregulation found in SLE. eCBs, of which the main representatives are *N*-arachidonoylethanolamine (or anandamide, AEA) and 2-arachidonoylglycerol (2-AG), are the more representative lipidic molecules of this family, which interact with type-1 (CB_1_) and/or type-2 (CB_2_) cannabinoid receptors [13,21]. These receptor targets, eCBs and their metabolic enzymes, form the so-called endocannabinoid system [22,23], which has manifold actions within the immune system, mainly expounding an anti-inflammatory effect [24]. Recently, it has been shown that 2-AG plasma levels are higher in SLE patients, compared to the general population, and correlate with a low disease activity, although this relationship has not been confirmed either for AEA nor for endocannabinoid-like molecules *N*-palmitoylethanolamine (PEA) and *N*-oleoylethanolamine (OEA). Moreover, the expression of diacylglycerol lipase (DAGL), the biosynthetic enzyme for 2-AG, is increased in peripheral blood mononuclear cells (PBMCs) from SLE patients compared to HD, thus suggesting that PBMCs are at least in part responsible for the production of this eCB [25]. Furthermore, in plasmacytoid dendritic cells (pDCs) of SLE patients, 2-AG is able to control the production of type 1 interferon (IFN) through CB_2_ activation [26].

The aim of the present study was to elucidate the potential role of 2-AG in modulating Th lymphocytes’ balance and cytokine production in SLE patients and matched healthy donors (HD).

## 2. Materials and Methods

### 2.1. Participants’ Characteristics

Participants with SLE and age- and sex-matched HD were consecutively recruited from the immuno-rheumatology outpatients clinic of Fondazione Policlinico Campus Bio-Medico, Rome, Italy. All the participants filled in and signed the informed consent. The study protocol was approved by the Internal Review Board (IRB) of Campus Bio-Medico University of Rome, protocol number 5416 oss. The study was conducted in compliance with International Conference on Harmonization Good Clinical Practice guidelines and the Declaration of Helsinki. Inclusion criteria included: (i) diagnosis of SLE according to the 2012 Systemic Lupus International Collaborating Cohort (SLICC) criteria [27]; (ii) serological disease activity, defined by anti-double strand DNA (anti-dsDNA) positivity and/or low plasma levels of complement, such as complement component 3 (C3) and/or C4 [28]; (iii) signed informed consent. Exclusion criteria were: (i) concomitant biological treatment (i.e., belimumab and rituximab) or in the last 12 months, (ii) cancer at enrolment or in the past five years, (iii) infectious disease at recruitment, (iv) corticosteroid pulse therapy in the last 6 months, (v) pregnancy or lactation, (vi) use of phyto-cannabinoids or synthetic cannabinoids in the 2 months before the enrolment. All the patients underwent rheumatological assessment, including systemic lupus erythematosus disease activity index (SLEDAI)-2k [29], and routine blood sampling, including autoantibodies and C3 and C4 levels measurements.

### 2.2. Peripheral Blood Mononuclear Cells (PBMCs) Isolation and Culture Conditions

Peripheral blood mononuclear cells (PBMCs) samples were collected from bloodstream from HD and SLE participants by Ficoll–Hypaque density-gradient centrifugation. The layer of mononuclear cells was collected, counted, and cultured in RPMI 1640 medium with 10% FBS supplemented with 2 mM glutamine and 50 mg/mL gentamycin. PBMCs were left untreated or were pretreated with 2-AG, purchased from Cayman Chemical Company, at increasing concentrations (0.01, 0.1, 1, and 10 μM) for 30 min, as reported elsewhere [30]. Cells were then stimulated with phorbol-12-myristate-13-acetate (50 ng/mL) and ionomycin (1 μg/mL) for 4 h. Brefeldin A (10 μg/mL) was also added. Where indicated, cells were pretreated for 30 min with selective antagonists of CB_1_ and CB_2_, AM251 and AM630, respectively, both used at a concentration of 200 nM. At the end of the treatments, samples were analyzed by flow cytometry.

### 2.3. Intracellular Cytokine Assay, CB Receptors Inhibition, and Flow Cytometry

To evaluate the percentage of lymphocytes producing IFN-ɣ and interleukin (IL-)4, cells were washed and stained with conjugated mAbs against human CD4, CD8, and CD3 (all from Miltenyi Biotec, Bergisch Gladbach, Germany) at +4 °C for 30 min. Cells were then fixed with 4% paraformaldehyde for 10 min and permeabilized by adding 0.5% saponin at room temperature for 30 min. Afterwards, cells were stained with cytokine-specific antibodies, as shown in Supplementary Table S1. For Treg and Th17 analysis, cells were first stained with conjugated mAbs against human CD4 and CD25 (Miltenyi Biotec, Miltenyi Biotec, Bergisch Gladbach, Germany) at +4 °C for 30 min. Cells were then fixed and permeabilized with the Foxp3 Fix/Perm kit (eBioscience, San Diego, CA, USA) according to manufacturer instructions, and additionally stained with mAbs against FoxP3 and IL-17 (Miltenyi Biotec, Miltenyi Biotec, Bergisch Gladbach, Germany, Supplementary Table S1). Acquisition was performed on CytoFLEX cytometer (Beckman Coulter, Brea, CA, USA), and data were analyzed using CytExpert software. Th17 cells were defined as CD4+ T cells producing IL-17, and Treg cells as CD4+CD25+FoxP3+ T cells. The gating strategy used to identify Treg cells, Th17 cells, and CD4+ lymphocytes expressing IL-4 is shown in Supplementary Figure S1. he vitality of CD4+ T lymphocytes was analyzed by flow cytometry using Viobility 488/520 Fixable dyes (Miltenyi Biotec). Briefly, PBMCs treated with 2AG and AM630 as previously described were collected and incubated with Viobility dye (1:100 diluition) for 15 min. After washing step, cells were stained with CD4 APC antibody (Miltenyi Biotec) for 30 min, and then samples were run on cytometer.

### 2.4. Cell Sorting

For the separation of CD4+ and CD8+ T lymphocytes, PBMCs from patients with SLE (N = 5) and HD (N = 5) were stained with CD4 APC and CD8 PE antibodies (Miltenyi Biotec), and cell sorting was performed with Cell Sorter MoFlo Astrios (Beckman Coulter). Purity of the enriched populations was greater than 98% in all experiments.

### 2.5. Total RNA Isolation and Quantitative Reverse Transcription Polymerase Chain Reaction (RT-qPCR) Analysis

The total mRNA was extracted using Relia Prep RNA cell Mini Prep System (Promega) according to the manufacturer’s protocol, and 200 ng of each RNA sample was reverse-transcribed using the SensiFast cDNA synthesis kit (BioLine).

Quantitative real-time PCR was performed on 7900HT Fast Real-Time PCR System (Applied Biosystems™) using specific 6-carboxyfluorescein (FAM)-labeled TaqMan assays for CB1 (Hs01038522_s1), CB2 (Hs00275635_m1) ribosomal protein L34 (Hs00241560_m1) as housekeeping genes. Each sample was loaded in duplicate with 5 ng of cDNA per well. Data were analyzed using the 2^-DCt method and reported as mean fold change in gene expression.

### 2.6. Statistical Analysis

Continuous variables are expressed as median (25th–75th percentile) or percentage, as appropriate. Normality of continuous variables has been assessed using the Shapiro–Wilk test, whereas differences between continuous variables have been analyzed using Wilcoxon test for paired data and Mann–Whitney test for unpaired data. Statistical analysis was performed using GraphPad Prism 7 (GraphPad Software, Inc., San Diego, Ca, USA).

## 3. Results

Twelve participants with SLE and twelve HD have been enrolled. The participants’ characteristics are summarized in Table 1.

All the SLE participants reported anti-nuclear antibodies positivity, ten of them showed low C3 and/or low C4 levels, six of them had anti-dsDNA antibodies or anti-Sm antibodies positivity, and four of them presented anti-phospholipids or anti-RNP antibodies positivity. None of the HD reported autoantibodies positivity or hypocomplementemia.

**Figure 1:**
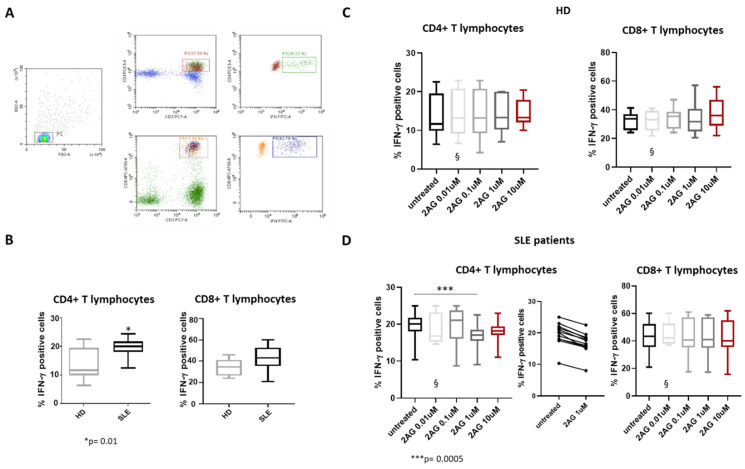
**Effect of 2-Arachidonoylglycerol (2-AG) on Th lymphocytes expressing IFN-ɣ.** (**A**) Representative dot plots of flow cytometry gating strategy used to identify IFN-ɣ-positive CD4+ T lymphocytes. (**B**) Statistical analysis of the percentage of CD4+ and CD8+ T lymphocytes expressing IFN-ɣ in healthy donors (HD) and SLE patients (n = 12 for each group). HD vs. SLE, * *p* = 0.01. (**C**,**D**) Analysis of the percentage of CD4+ and CD8+ T lymphocytes expressing IFN-ɣ after treatment with 2-AG at concentrations of 0.01, 0.1, 1 and 10 μM in HD (C) and SLE patients (D). Data are represented as box plots displaying medians, 25th and 75th percentiles as boxes, and 10th and 90th percentiles as whiskers. § = data obtained from 5 HD and SLE patients representative of entire population. *** *p* = 0.0005.

As reported in Figure 1, the SLE participants showed a higher percentage of CD3^+^CD4^+^IFN-ɣ ^+^ (namely Th1 lymphocytes) compared with HD (*p* = 0.01).

No statistically significant changes in the percentage of CD3+CD8+IFN-ɣ+ were found between the two groups (Figure 1B).

Moreover, in the SLE patients, the addition of 1 μM of 2-AG induced a decrease in IFN-ɣ expression compared to untreated (*p* = 0.0005), whilst HD treatment with increasing doses of 2-AG did not provide any difference in IFN-ɣ expression from PBMCs compared to the untreated condition (Figure 1C,D). Regarding the CD8+ T lymphocyte subset, 2-AG had no effect on the percentage of cells producing IFN-ɣ in both HD and SLE patients (Figure 1C,D). To explore the differential response to 2-AG treatment between HD and SLE patients, we assessed the expression of CB receptors by real-time PCR. In freshly isolated CD4+ T lymphocytes and CD8+ T lymphocytes from HD and SLE patients, the gene expression of CB_1_ and CB_2_ did not change in a statistically significant manner (Supplementary Figure S2).

**Figure 2:**
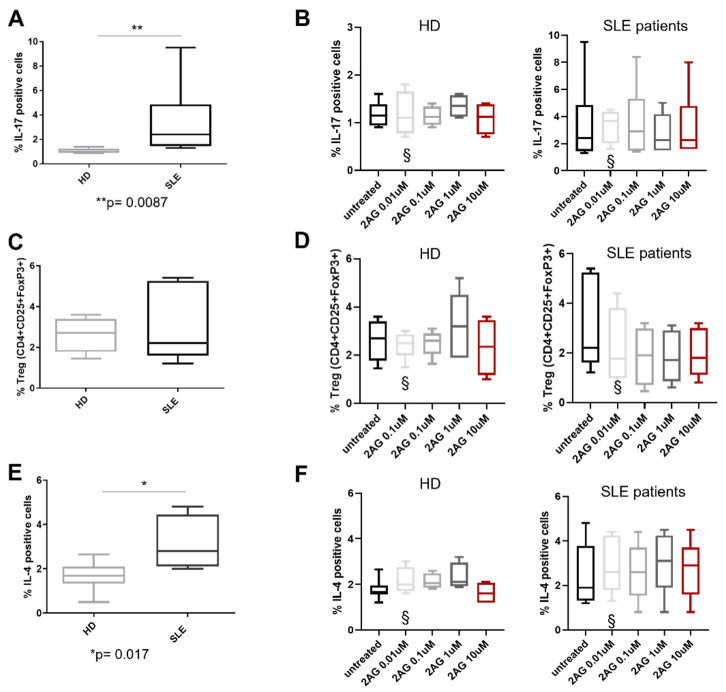
**Effect of 2-Arachidonoylglycerol (2-AG) on Th17, Treg, and Th2 lymphocytes.** (**A**,**C**,**E**) Percentage of Th17, Treg, and Th2 lymphocytes in HD and SLE patients analyzed by flow cytometry (n = 12 for each group). *p* < 0.05. (**B**,**D**,**F**) Percentage of Treg/Th17 cells and lymphocytes expressing IL-4 after treatment with 2-AG at concentrations of 0.01, 0.1, 1 and 10 µM in HD and SLE patients. § = data obtained from 5 HD and SLE patients representative of entire population.

As reported in Figure 2, the SLE participants showed a higher percentage of CD3^+^CD4^+^IL17A^+^ (namely Th17 lymphocytes) compared with HD (*p* = 0.008, Figure 2A).

On the contrary, no difference in the CD3^+^CD4^+^FoxP3^+^ (namely Treg lymphocytes) between the two groups has been reported (Figure 2C). The percentage of Th2 lymphocytes (CD3^+^CD4^+^IL4^+^ cells) was increased in patients affected by SLE compared to HD (*p* = 0.04, Figure 2E). Furthermore, treatment with increased concentrations of 2-AG did not induce any modification in IL-17A, FoxP3, or IL-4 expression in Th17, Treg, and Th2 lymphocytes, respectively, from both SLE participants and HD (Figure 2). 

**Figure 3:**
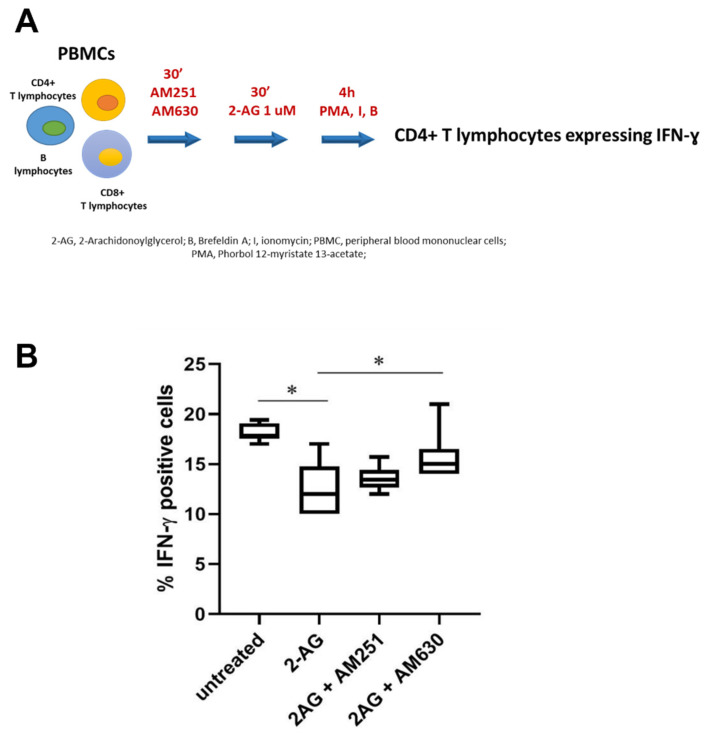
**Effect of CB_1/2_ antagonists on Th1 lymphocytes expressing IFN-**ɣ **in SLE patients. (A)** Experimental design. PBMCs isolated from 6 SLE patients were pretreated for 30 min with selective antagonists of CB_1_ and CB_2_ receptors, AM251 and AM630, respectively (200 nM). Then, 2-AG (1 μM) was added to the culture and cells were analyzed by flow cytometry as previously described. (**B**) Flow cytometry analysis of the percentage of Th1 lymphocytes expressing IFN-ɣ after treatment with AM251 and AM630 CB antagonists and 2-AG in SLE patients (*n* = 6). * *p* < 0.05.

In order to elucidate which cannabinoid receptor might be involved in the modulation of IFN-ɣ expression, PBMCs have been exposed to 2-AG in the presence of AM251 (a CB_1_ antagonist) or AM630 (a CB_2_ antagonist, Figure 3). For this latter experiment, a subset of six patients were randomly selected from the twelve overall patients.

Our results show that treatment with 1 μM of 2-AG+AM630 significantly increased IFN-ɣ expression in Th1 lymphocytes of SLE patients compared to Th1 lymphocytes treated only with 2-AG (*p* = 0.03, Figure 3B), confirming the involvement of CB_2_ antagonist. On the contrary, IFN-ɣ expression in Th1 lymphocytes of SLE patients does not significantly differ between untreated cells and cells treated with 1 μM of 2-AG+AM251.

Furthermore, the analysis of dead cells by flow cytometry showed that 2-AG alone or in combination with AM630 did not affect the vitality of CD4+ lymphocytes from SLE patients (Supplementary Figure S3).

## 4. Discussion

To the best of our knowledge, it is the first time that the ability of 2-AG in actively modulating the production of IFN-ɣ from Th1 lymphocytes of SLE patients has been documented.

This observation extends the previous knowledge on the effects of 2-AG on IFN production in SLE. Indeed, it has already been demonstrated that 2-AG may modulate the production of type 1 IFN in pDCs from SLE patients [26], but no information was as yet available about its potential effect on type 2 IFN (namely IFN-ɣ). Here, it has been shown analogously to pDCs, in which 2-AG activity reduces interferon-related genes expression and 2-AG modulates IFN-ɣ production in lymphocytes, thus reflecting its broader range of action on the immune system and IFN types. The functional and epigenetic commitment of the different immune cell lines may be the reason behind these different IFN effects of 2-AG. IFNs, in fact, underpin important immunologic activities against viral infections and tumors and in modulating adaptive immune responses, playing a key role in many autoimmune diseases [31,32]. Furthermore, the degradation of 2-AG by monoacylglycerol lipases might present inter-individual variability, as shown in pDCs. This difference in the activity of these enzymes, namely ABHD6 and ABHD12, might entail a difference in the amplitude on the IFN-ɣ modulation of 2-AG between patients. In addition, one of the degradation products of 2-AG is COX; therefore, a high rate of enzymatic activity might further counteract the immune modulatory effects of 2-AG through the production of this pro-inflammatory molecule and possibly explain the limited effects of higher concentrations of 2-AG in our experimental setting.

2-AG is a major endocannabinoid that primarily acts as a ligand at CB_1_ and CB_2_ receptors, thus modulating immune cells activation and effector functions. 2-AG was found in the synovial fluid of patients with osteoarthritis and rheumatoid arthritis (RA), while the presence of this molecule was not detected in healthy donors [33]. Moreover, in vitro studies on the effect of synthetic cannabinoids CP55,940 and WIN55,212-2 in RA synovial fibroblast showed that these compounds reduced the production of pro-inflammatory cytokines, as well as the release of matrix metalloproteinases [34]. Additionally, a neuroprotective function of 2-AG in experimental autoimmune encephalomyelitis (EAE), an animal model of multiple sclerosis (MS), has been proposed [35]. In 2-AG-treated animals, a shift to M2 macrophages differentiation has been documented, suggesting possible involvement of 2-AG in the regulation of the inflammatory milieu, an issue that deserves further investigation.

Our results showed that 2-AG did not influence the Treg/Th17 axis. However, it has been found that 2-AG treatment decreased Th17-associated cytokines expression in mice [36]. Furthermore, Chiurchiù and colleagues showed that bioactive lipids belonging to the ALIAmides family enhanced *de novo* generation of regulatory T cells from CD4-naive T cells [37]. It is possible that an in vitro expansion of Treg and Th17 cells is needed to better explore the effect of 2-AG on these cell subsets’ distribution. 

There is growing evidence that IFN-ɣ production is a critical step in SLE pathogenesis. The relationship between IFN-ɣ and autoimmunity development is still poorly understood, but it is well established that type 2 IFN production is able to promote T cell differentiation and, in B cells, class switch towards more pathogenetic autoantibodies [38]. Unsurprisingly, the mutation in the *Roquin^san/san^* lupus mice model, with increased IFN-ɣ signaling, is characterized by follicular Th (fTh) cells and enhanced autoantibodies secretion [39]. Moreover, in another mouse model of SLE, deficiency in IFN-ɣ receptors leads to the reduction in antinuclear antibodies reactivity and lowered IgG2c and IgG2b autoantibodies production [40]. Type 2 IFN serum concentrations are increased in SLE patients compared to HD, and they correlate with disease activity [41]. Furthermore, elevated levels of IFN-γ were detected in serum from preclinical SLE patients, suggesting a possible early role of this cytokine in SLE pathogenesis [42]. 

The increased expression of this cytokine induces the production of soluble B lymphocyte stimulator (sBLyS) by monocytes and macrophages, thus indirectly increasing activation and maturation of B lymphocytes [43]. Furthermore, IFN-ɣ +874 T/A polymorphism is associated with an increased risk of SLE development in the Chinese Han population [44], and IFN-related genes play a pivotal role in SLE pathogenesis, especially IFN-ɣ and IFN-ɣ inducible GBP1 gene in the early stages of the disease [45]. Moreover, serum and urine concentrations of IFN- ɣ might be implicated in lupus nephritis and might represent a promising biomarker of this manifestation [46,47,48]. IFN-ɣ might also be implicated in central nervous system alterations observed in SLE [49]. Recently, it has been demonstrated that IFN-ɣ production by T-bet^+^ CD4^+^ cells is regulated by metabolic regulators, such as fatty acid synthesis inhibitors [50]. In keeping with our data, Kaplan and colleagues showed that, in splenocytes derived from CB_1_^−/−^/CB_2_^−/−^ mice, treated in vitro with 2-AG, the IFN-ɣ secretion was reduced [51]. Here, we show that the 2-AG effect on IFN-ɣ expression in CD4^+^ cells is strictly related to CB_2_.

No significant difference in CB1 and CB2 mRNA expression was found in both CD4+ and CD8+ lymphocytes from a subgroup of SLE participants and HD. Nevertheless, further studies, with a specific enrolment strategy, are required to fully demonstrate possible differences in CB1 and CB2 protein expression.

CB_2_ is abundantly expressed by immune cells, where it exerts relevant anti-inflammatory effects [44,52]. Therefore, our present data seem to support the hypothesis that the pharmacological modulation of CB_2_ may represent a new therapeutic strategy to address both type 1 and type 2 IFN responses.

In the last few decades, the possible use of plant-derived or synthetic cannabinoids in clinical practice for the treatment of several disorders has been proposed. For instance, rimonabant, the first inverse agonist of CB_1_ approved for obese patients, was withdrawn from the market due to adverse effects related to the central nervous system, including depression and suicidal ideation. However, it has recently been shown that the chronic administration of rimonabant in rats was not associated with development of adverse psychiatric phenotypes, suggesting that the analysis of a patient’s comorbidity, such as obesity, is fundamental to prevent this (and other possible) side effect [53]. Moreover, the efficacy and safety of lenabasum, a novel oral CB_2_ agonist, is currently under investigation in multiple autoimmune and fibrotic diseases, including SLE and systemic sclerosis [54]. Unfortunately, in the latter disease, lenabasum failed to meet the primary endpoint [54].

The present study suffers from some limitations. Firstly, we evaluated only CD4+ and CD8+ IFN-ɣ-producing cells, but a few other immune cell types may produce this cytokine, especially natural killer cells. Furthermore, only intracellular production of IFN-ɣ has been assessed. Finally, in this specific experimental setting, we were unable to fully explain the observed effect on CD4+ T cells from SLE patients.

Despite these limitations, our work is the first to describe an anti-inflammatory role of the 2-AG/endocannabinergic axis on T cells, thus expanding our knowledge on the possibility to target this axis for future therapies of SLE patients.

## 5. Conclusions

Our data expand the horizons of 2-AG involvement in SLE immunopathogenesis. 2-AG is actually able to affect both type 1 and type 2 IFNs in this disease. These findings support the important role of bioactive lipids in autoimmune diseases. 

## Figures and Tables

**Table 1:** Participants’ characteristics. SLEDAI: systemic lupus erythematosus disease activity index; dsDNA: double strand DNA; Sm: Smith; RNP: ribonucleoprotein; C: complement.

Variable	SLE Participants (n = 12)	Healthy Donors (n = 12)
Age (years)	42 (34.5–54.25)	44 (33–57.5)
Disease duration (months)	123 (38.25–144.5)	NA
SLEDAI-2k	2 (2–2.75)	NA
Antinuclear antibodies positivity (n)	12	0
Anti-dsDNA antibodies positivity (n)	6	0
Anti-Sm antibodies positivity (n)	6	0
Anti-phospholipids antibodies positivity (n)	4	0
Anti-RNP antibodies positivity (n)	4	0
Low C3 or C4 levels (n)	10	0

## Data Availability

Data is contained within the article and Supplementary Materials.

## Supplementary Materials

The following supporting information can be downloaded at: https://www.mdpi.com/article/10.3390/biomedicines10071675/s1, Figure S1: Representative dot plots of peripheral blood lymphocytes from one SLE patient showing the gating strategy to identify Treg cells (A), Th17 cells (B) and CD4+ T lymphocytes expressing IL-4 (C), all within the P2 population; Figure S2: Gene expression of CB1 and CB2 in CD4+ (A, B) and CD8+ (C, D) cells of SLE patients compared to healthy donors. Values are reported as fold change in gene expression over ribosomal protein L34. Data are shown as means ± SEM, n = 5 samples per group. No significant difference using the Mann-Whitney test; Figure S3: Effect of 2-AG and CB2 inhibitor AM630 on cell vitality. Representative flow cytometry dot plots showing the percentage of dead CD4+ T lymphocytes from SLE patients (n = 5) after treatment with 2-AG alone and in combination with AM630; Table S1: antibodies used for flow cytometry analysis. IL: interleukin; IFN: interferon.
