# Supplementary material for: 2-Arachidonoylglycerol Reduces the Production of Interferon-Gamma in T Lymphocytes from Patients with Systemic Lupus Erythematosus
Source: Biomedicines. 2022 Jul 12;10(7):1675. doi: 10.3390/biomedicines10071675 (PMC9312521; doi:10.3390/biomedicines10071675)
Supplement: Supplementary file 1 [file biomedicines-10-01675-s001.zip › biomedicines-1708186-supplementary.pdf]

Supplementary Materials

Supplementary Table S1. antibodies used for flow cytometry analysis. IL: interleukin; IFN: interferon.

Antibody	Diluition
CD4 PercP Vio 700	1:100
CD8 APC Vio 770	1:100
CD3 PE Vio 770	1:100
IL-4 PE	1:50
IFN-γ FITC	1:50
FoxP3 PE	1:50
IL-17A APC	1:50
CD4 APC	1:100

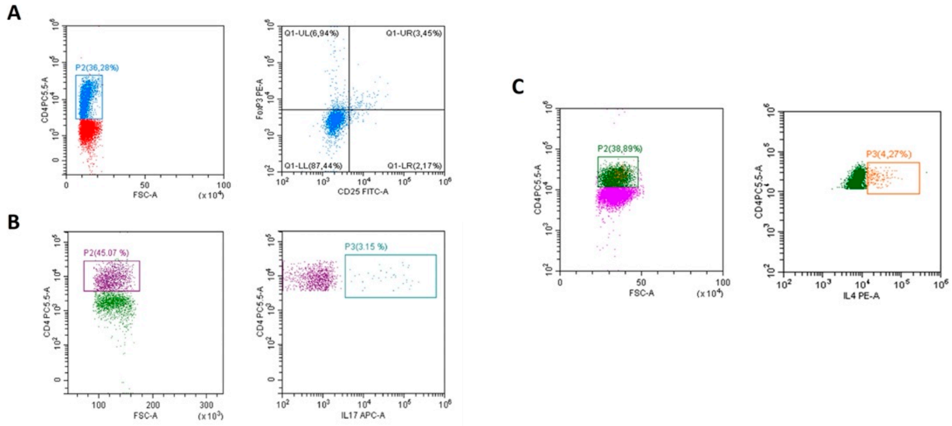

Supplementary Figure S1. Representative dot plots of peripheral blood lymphocytes from one SLE patient showing the gating strategy to identify Treg cells (**A**), Th17 cells (**B**) and CD4+ T lymphocytes expressing IL-4 (**C**), all within the P2 population.

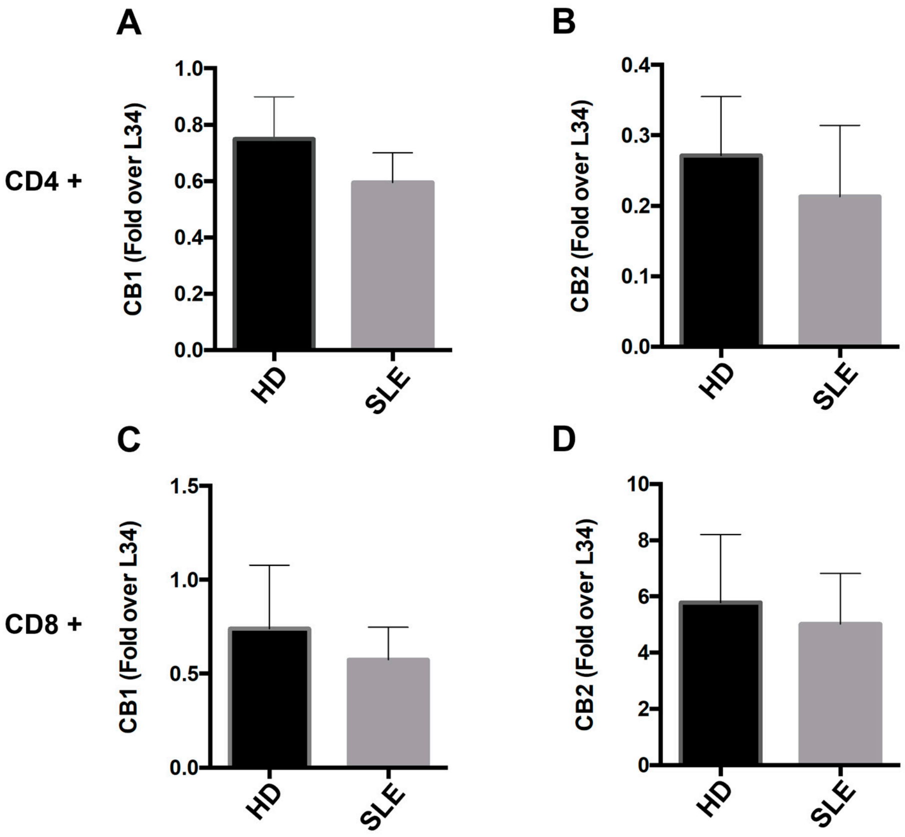

Supplementary Figure S2. Gene expression of CB_1 and CB_2 in CD4+ (**A**,**B**) and CD8+ (**C**,**D**) cells of SLE patients compared to healthy donors. Values are reported as fold change in gene expression over ribosomal protein L34. Data are shown as means ± SEM, n = 5 samples per group. No significant difference using the Mann-Whitney test.

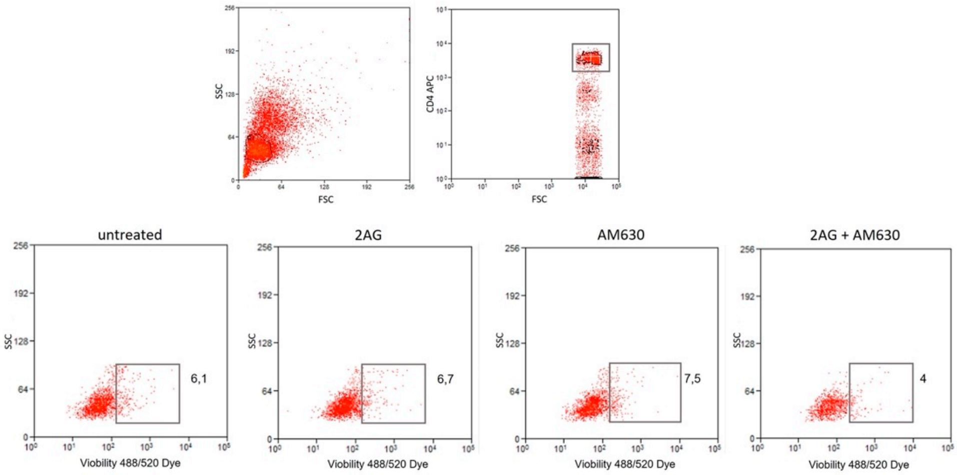

Supplementary Figure S3. Effect of 2-AG and CB_2 inhibitor AM630 on cell vitality. Representative flow cytometry dot plots showing the percentage of dead CD4+ T lymphocytes from SLE patients (n = 5) after treatment with 2-AG alone and in combination with AM630.
